# Adult Sex Ratio as a Demographic Feedback Linking Mating Systems, Parental Care, and Evolution

**DOI:** 10.1002/advs.202522134

**Published:** 2026-02-09

**Authors:** Tamás Székely, Oscar G. Miranda

**Affiliations:** ^1^ Milner Centre for Evolution Department of Life Sciences University of Bath Bath UK; ^2^ HUN‑REN‑DE Reproductive Strategies Research Group Department of Evolutionary Zoology and Human Biology University of Debrecen Debrecen Hungary; ^3^ Department of Ethology Eötvös Loránd University Budapest Hungary

**Keywords:** animal behavior, conservation, reproduction, sexual size dimorphism

## Abstract

Behavioral ecology is shifting from static models of sex‐role evolution to dynamic frameworks in which demography and behavior form coupled feedback systems. Here, we outline a unifying approach developed at the University of Bath—the *demographic feedback model*—that links individual decisions to population structure through the adult sex ratio (ASR), a measurable demographic variable that integrates survival, maturation, and reproduction. Across species, sex‐biased survival and remating opportunities generate a recursive feedback between mating systems, parental care, and sexual dimorphism. This feedback, in turn, shape population viability and conservation outcomes. By embedding ASR monitoring within long‐term field studies and comparative phylogenetic analyses, our research reveals how demographic distortions propagate through social behavior, and how behavior feeds back to restructure demography. The resulting feedback framework connects behavioral ecology, life‐history theory, and conservation demography under a single logic: populations evolve not through linear causation, but through nested feedbacks linking genes, ecology, and behavior. We identify four key frontiers—cross‐taxon field protocols, causal comparative inference, nested‐feedback theoretical modelling, and integration with biodiversity conservation practice—that will define the next generation of research. Recognizing behavior as both a product and a driver of demographic structure opens a path toward a genuinely recursive theory of evolution.

## Graphical abstract

Breeding systems are some of the most diverse social behavior, and our team is investigation the evolutionary causes of this diversity. This review summarises our research carried out at the University of Bath. We argue that demographic components of wild populations, especially the adult sex ratio, plays a key role driving breeding system variation. We critically overview this research field, and recommend future investigations.

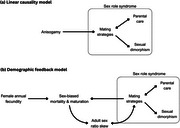

“*If a subject is already receiving a great deal of attention, if it has a glamorous aura, if its practitioners are prize‐winners who receive large grants, stay away from that subject*.”

(Wilson, 2013)

## 1 Introduction

To challenge an established paradigm, a budding scientist may not always study glamorous organisms, charismatic megafauna or fashionable theories. As E. O. Wilson advised young biologists, ‘*if a subject is already receiving a great deal of attention… stay away from that subject*’ [1]. The study of behavioral ecology at the University of Bath offers a vivid example of how this philosophy can flourish. When the first author began his career at Bath in 2000, offspring desertion was a marginal topic, well described in natural history, yet theoretically fragmented and overshadowed by the dominant focus on sexual selection and sex allocation [2]. Yet this neglected corner of evolutionary biology, focused on offspring desertion, confronted a fundamental problem: what determines how males and females divide their parental efforts, and why do some parents care for their young while others do not?

Understanding how these questions took shape requires examining the intellectual and methodological landscape from which they emerged. Late twentieth‐century behavioral ecology was guided by elegant but incomplete paradigms that rarely connected individual behavior with population dynamics, ecology, or conservation [3, 4, 5, 6]. Bridging these gaps demanded more than new models: it required mindsets grounded in long‐term fieldwork, comparative analyses, and bold conceptual thinking. These early experiences laid the foundation for an integrative vision in which behavior, ecology, and evolution are studied as interacting systems linked by feedbacks across multiple scales.

This shift also mirrored a wider transformation in twenty‐first‐century biology, from specialization to synthesis [7]. Over recent decades, science has increasingly sought to connect disciplines, methods, and applications, uniting developmental biology, ecology, physiology and conservation within shared conceptual frameworks [8, 9]. This integrative turn has broadened the scope of evolutionary theory while enhancing its relevance to global challenges such as biodiversity loss and environmental change. The study of sex roles became one arena in which this synthesis proved especially fruitful, revealing how demographic and ecological feedback shape biodiversity and inform its conservation.

This review traces how these ideas evolved into a coherent framework linking field observations, theory building, and conservation practice. It highlights the collaborative and interdisciplinary culture fostered at the University of Bath, and through its international partnerships, which allowed a once‐unfashionable topic to mature into an influential research programme. Here, we explore this trajectory in three sections: (1) the origins of an integrative vision of sex‐role research; (2) the development and expansion of the feedback framework, with particular emphasis on the role of the adult sex ratio (ASR); and (3) its application to biodiversity conservation and future challenges for behavioral ecology. For terminology and detailed accounts of this research programme, see Székely [10, 11, 106]. This synthesis emerged from a collaborative literature review conducted by both authors during the second author's doctoral research at the University of Bath, which focuses on demographic feedbacks linking adult sex ratio, mating systems, parental care, and evolutionary dynamics.

## 2 Seeds of an Integrative Vision

**FIGURE 1:**
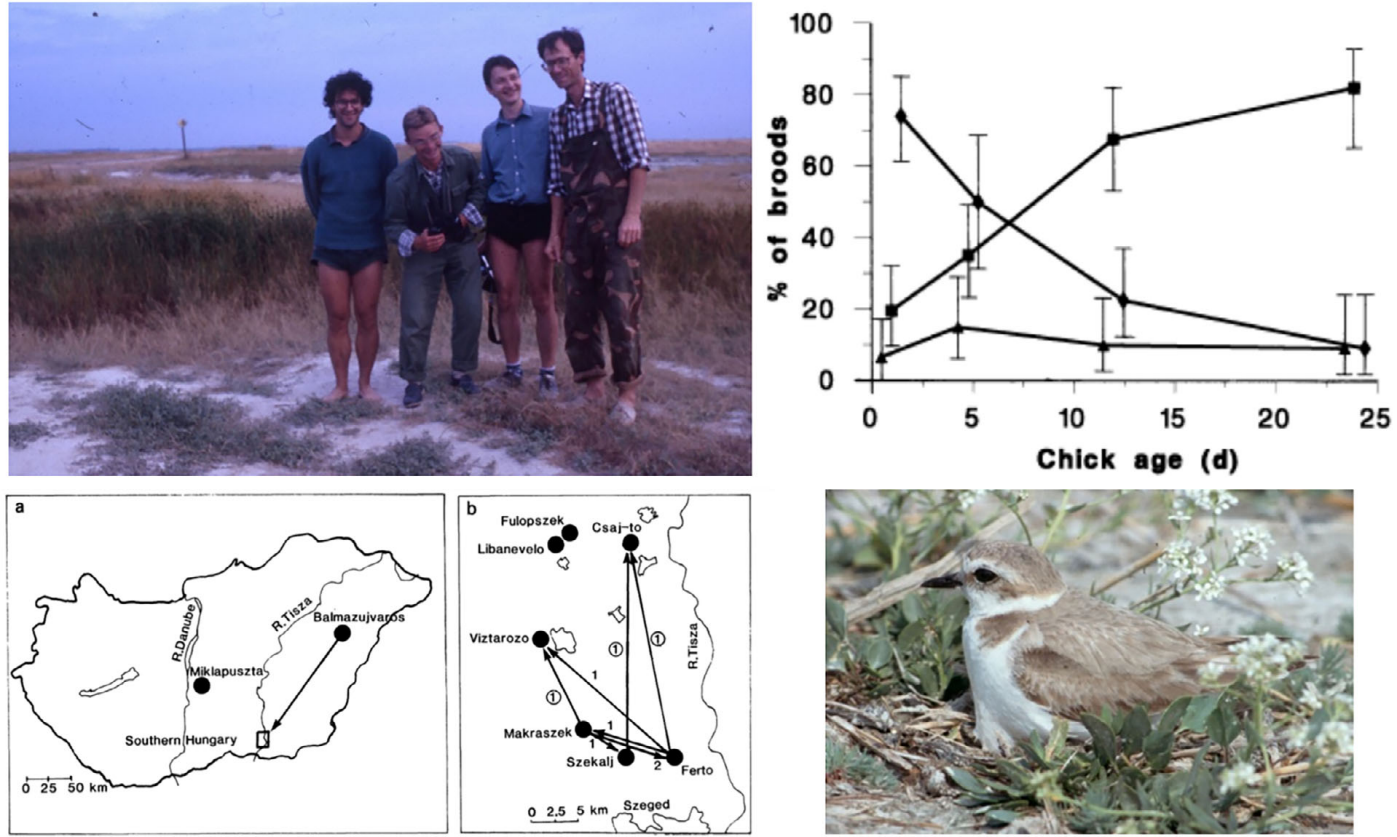
Initial years of Kentish plover research in Hungary (1988 – 1994). The research team (top left: András Liker, Sándor Kovács, László Lisztes, Tamás Székely, from left to right). Brood care (top right) and movements of adults (bottom left) Kentish plovers in Hungary [18], copyright pending). Female Kentish plover on her nest (bottom right, credit: G. Lendvai).

The origins of our integrative approach trace back to Tamás Székely's formative years as a field biologist in Hungary (Figure 1). His early studies on the distribution and social behavior of birds in relation to forest succession [12] revealed a lasting interest in how ecological context shapes animal societies. While investigating the flocking behavior of passerine birds, he showed that the two major hypotheses of group living—the antipredation and foraging‐efficiency hypotheses—could explain mixed‐species flocks, whereas territorial maintenance appeared more important in single‐species groups [13]. These early insights sowed the seeds of a lifelong curiosity about how ecological factors structure social systems.

As the research began to focus on the social dynamics of the sexes, clear behavioral differences between males and females within species came to light. Early work suggested that variation in foraging behavior depended largely on ecological guild and niche use, rather than on sex—an idea that foreshadowed later concepts of sex‐specific strategies [14]. Collaboration with Alasdair Houston, who pioneered dynamic optimization modelling together with John McNamara, allowed to explore the trade‐offs between antipredator vigilance and feeding efficiency [15]. The quantitative modelling skills gained through these studies led to the recognition that a proper understanding of animal behavior requires theoretical models capable of describing decision‐making across diverse ecological contexts.

From 1988 onward, attention turned increasingly to parental care. A key moment was the decision to study the breeding behavior of a small, inconspicuous shorebird—the Kentish plover (*Charadrius alexandrinus)* [16, 17]. Fieldwork in Hungary revealed that this species is ideal for investigating reproductive trade‐offs, because every stage of its life cycle, from eggs to adults, can be readily observed and measured in the field (Figure 1). The first years of study revealed striking variation in parental and mating behavior in both sexes. The work further showed that plovers frequently change partners, that the timing of brood desertion shifts seasonally, and that these patterns likely reflect changing mating opportunities [18, 19, 20, 21]. In other words, the speed with which each sex remates varies with the population sex ratio—an insight that introduced the then‐unconventional idea that mating opportunities mediate decisions to remain with the brood or desert the young.

Parallel to fieldwork, two complementary research directions deepened the understanding of parental behavior. One line of inquiry applied newly emerging phylogenetic comparative methods to shorebirds—the broader clade that includes plovers, sandpipers, curlews, and related taxa [22, 23]. These studies suggested that biparental incubation and independence from feeding the young facilitate offspring desertion by females, transforming what had initially appeared to be an anomaly in Kentish plovers into a recurrent evolutionary pattern among shorebirds.

The second line of research, developed through collaborations with theoreticians, produced a mathematical framework that linked mating opportunities with parental and mating strategies in a recursive feedback system. Initial game‐theoretic models focused on two reproductive stages and demonstrated that incorporating a feedback between caring and remating could generate mixed‐strategy equilibria—outcomes that are impossible under traditional static models [24]. A subsequent model expanded these ideas to biologically more realistic scenarios by modelling male and female behavior across an entire breeding season at the population level [25].

These empirical and theoretical strands converged in 1998 at a symposium in Erice (Italy), where an emerging vision of breeding systems was articulated: mating and parental behavior should no longer be studied in isolation, but as components of a feedback system shaped by frequency‐dependent interactions within an ecological context [26]. This moment marked a conceptual turning point—a move beyond the linear causality of Robert Trivers’ [4] parental‐investment model toward a recursive, dynamic view of sex‐role evolution. Rather than a culmination, it provided a map: a unifying framework that would guide subsequent research. When the group was established at the University of Bath in 2000, this idea became the foundation for a long‐term empirical programme aimed at testing, refining, and expanding theoretical predictions using data drawn from diverse species across the globe.

## 3 From Unidirectional Causality to Feedback Systems

**FIGURE 2:**
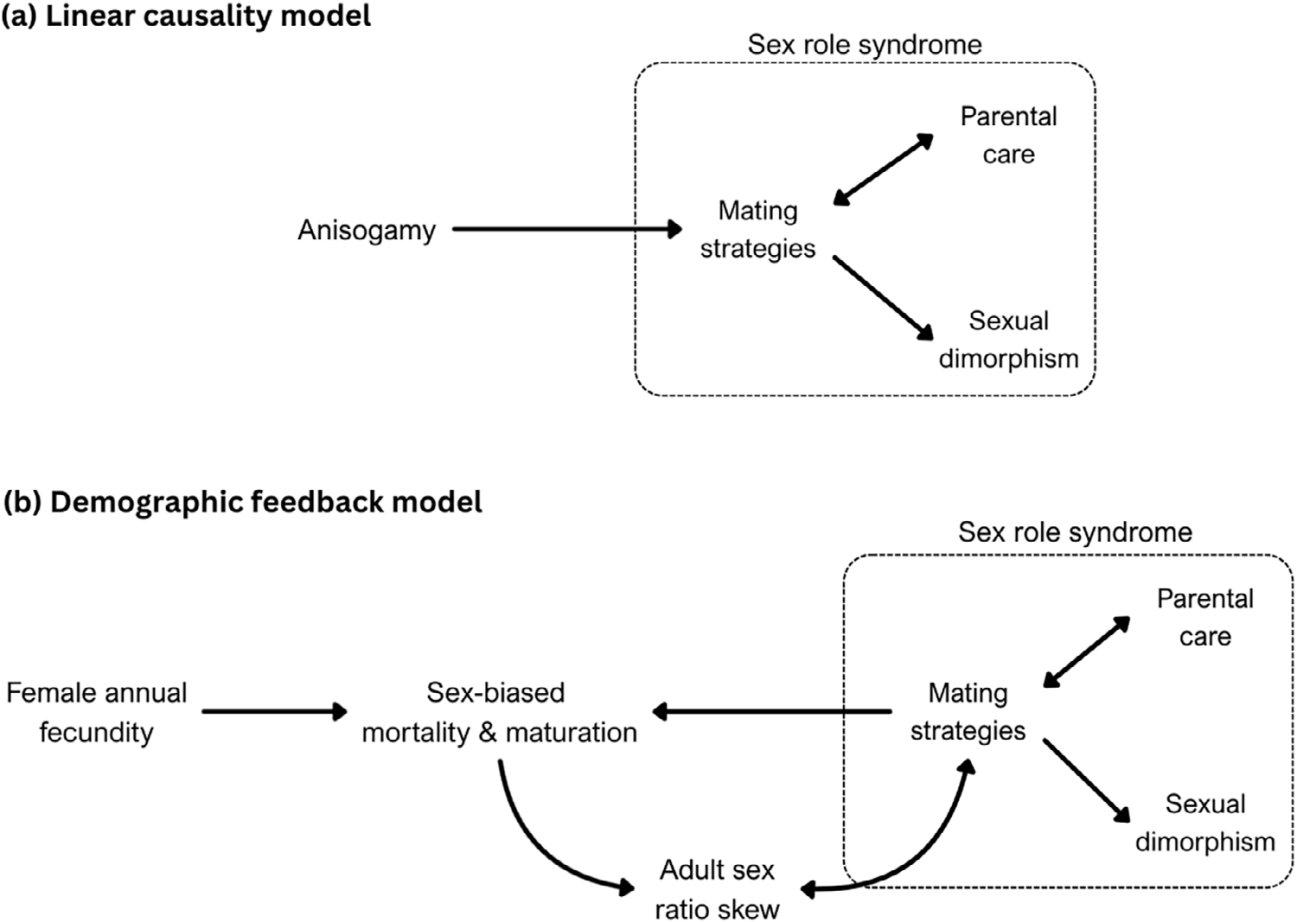
Conceptual shift from linear causation to demographic feedback in sex‐role evolution. Classical models (a) depict sex roles as downstream consequences of anisogamy, with mating strategies shaping parental care and sexual dimorphism [94]. In contrast, the demographic feedback model (b) places demographic processes at the core of sex‐role evolution. Female annual fecundity generates sex‐biased mortality and maturation, producing skewed adult sex ratios (ASR) that feedback on mating strategies and associated sex‐role traits [59, 63, 95]. Through reciprocal effects on survival, maturation, and competition, sex roles and demography coevolve in a closed feedback system.

The conceptual pivot of this framework is a shift from one‐way causality to reciprocal feedback, formalized here as a demographic feedback model of sex‐role evolution (Figure 2). Twentieth‐century narratives in behavioral evolution tended to run in a single direction. In the classic Darwin–Bateman cascade, anisogamy biases sexual selection, which in turn produces sex‐specific differences in mating and parental roles [27, 28]. An alternative view holds that ecological competition for resources generates dimorphism, which then channels behavior [29, 30]. Both explanations are elegant but incomplete.

In the demographic feedback model, mating strategies, caring strategies, and sexual dimorphisms are co‐determined by the mating‐opportunity structure of populations (Figure 2). Because behavior alters survival, partner availability, and the timing of breeding, it also feeds back to reshape the demographic structure of populations [26]. The same logic applies to phenotype: dimorphism emerges from current social and ecological conditions and, in turn, modifies how individuals compete for mates, choose breeding partners, and care for their young [31]. In short, behavior, phenotype, and demography form a coupled, frequency‐dependent system.

A collaborative research environment at the Biodiversity Lab of University of Bath—later integrated into the Milner Centre for Evolution—provided the setting in which this idea matured. The rhythm of the lab became recursive: models refined the questions; experiments and long‐term fieldwork tested them; and comparative phylogenetic analyses asked whether the resulting patterns held across clades [32, 33, 34]. Within this paradigm, focused on breeding systems, three interconnected advances crystallized: (a) demonstrating feedback loops both empirically and theoretically; (b) transforming the abstract notion of “mating opportunity” into a measurable demographic parameter, the adult sex ratio (ASR); and (c) extending the feedback framework to biodiversity conservation, making it directly relevant to the protection and management of wild populations.

### 3.1 Demonstrating Feedback Relationships

In the early 2000s, the research began to dissect how mating and parental care respond to ecological and social conditions—and how those responses, in turn, reshape the very context from which they arise. It further revealed that reproductive decisions often anticipate future trade‐offs.

Research on Kentish Plovers uncovered a direct behavior–demographic link: greater remating opportunities for females were not driven by biased sex allocation at laying or hatching that produced more males than females, but by higher mortality of female chicks soon after hatching [21, 33, 35]. This finding provided the first empirical evidence that demographic asymmetries—here, a skew in juvenile survival—can reshape adult mating dynamics and, ultimately, sex roles. Parallel work on magnificent frigatebirds (*Fregata magnificens*) revealed strategic negotiation within pairs: females adjusted their parental care according to their mate's behavior and the likelihood of desertion, providing rare evidence of real‐time behavioral rules tuned to reproductive opportunity [36].

Building on these species‐specific insights, the next step was to test whether the same behavioral–demographic feedback could be generalized to other systems. The Eurasian penduline tit (*Remiz pendulinus*) provided an ideal model: within a single population, pairs may exhibit male‐only care, female‐only care, biparental care, or even joint desertion [37, 38, 39, 40]. This extraordinary flexibility created a unique opportunity to examine, within a single species, how care and mating decisions both shape, and are shaped by, the surrounding social environment.

Thus, the Bath research group linked male care, mate guarding, and extra‐pair paternity as coordinated decisions under sexual competition, exposing a direct trade‐off between caring for current offspring and securing future mating opportunities [41, 42, 43]. Follow‐up field studies revealed that male penduline tits often deserted early clutches, when remating opportunities were abundant, but provided care later in the season as those opportunities declined [44]. Females, by contrast, remained consistent in their behavior across clutches. Together, these patterns demonstrated a real‐time feedback between demography and behavior: shifts in mating‐opportunity structure altered male decisions to care, and those decisions—by changing offspring survival and partner availability—fed back to reshape the very mating environment that selected for them [45, 46, 103].

Cross‐species experiments extended the feedback framework beyond single populations. Mate‐removal experiments in three closely related plovers—Kittlitz's, white‐fronted, and Kentish—showed that remating time and courtship effort vary with each species’ social system. In male‐biased populations, females remated sooner, whereas in species with more balanced adult sex ratios and higher parental cooperation, remating rates were similar between the sexes [47]. Even pair‐bond stability responded to these demographic conditions: newly formed pairs either persisted or collapsed depending on whether original mates returned, demonstrating that behavioral choices continually reshape the pool of available partners [48].

Together, these studies demonstrated the full cycle of feedback in action: demographic structure shapes mating and care decisions; those behavioral adjustments alter survival, pair stability, and partner availability; and these changes, in turn, feedback to redefine the demographic landscape from which the behaviors arise.

### 3.2 Quantifying Opportunity via Adult Sex Ratio

A central advance of this framework was the replacement of the abstract notion of “mating opportunity” with a measurable demographic variable: the adult sex ratio (ASR)—the proportion of males in the adult population [49, 50]. Early demographic modelling in a Turkish population of Kentish plovers revealed an extremely male‐biased ASR that matched experimental evidence for female‐biased mate availability, identifying ASR as a plausible proximate driver of sex‐role reversal in this species [51]. From the outset, ASR offered something conceptually new: it captures both cause and consequence [31]. Because ASR reflects demographic processes while simultaneously constraining social behavior, it can act as both a predictor and a response within the feedback system.

Time‐series analyses soon clarified why ASR, rather than the operational sex ratio (OSR), provides a better measure of long‐term mating opportunity. The OSR reflects short‐term fluctuations in the ratio of sexually active males and females—a volatile, event‐bound snapshot of breeding activity [52]. In contrast, ASR integrates survival, maturation, and recruitment over multiple seasons, representing the slow‐moving demographic substrate upon which operational ratios fluctuate [53, 54]. This stability makes ASR a more powerful and interpretable proxy for evolutionary and ecological inference.

Once established conceptually, ASR became a practical tool for both field demography and comparative phylogenetics. Methodological reviews outlined best practices for estimating ASR in the wild—through detection‐based counts, sex‐structured mark–recapture, and demographic reconstruction—allowing sex structure to enter mainstream population models [49, 55]. Empirical studies of plover populations, combined with large‐scale comparative datasets, revealed that juvenile survival—rather than adult survival—can be the primary driver of ASR skew [56, 57]. A central message of these studies was that ignoring the sex‐specific differences in demography can lead to biased estimates of population viability, directly linking ASR to biodiversity conservation [58].

**FIGURE 3:**
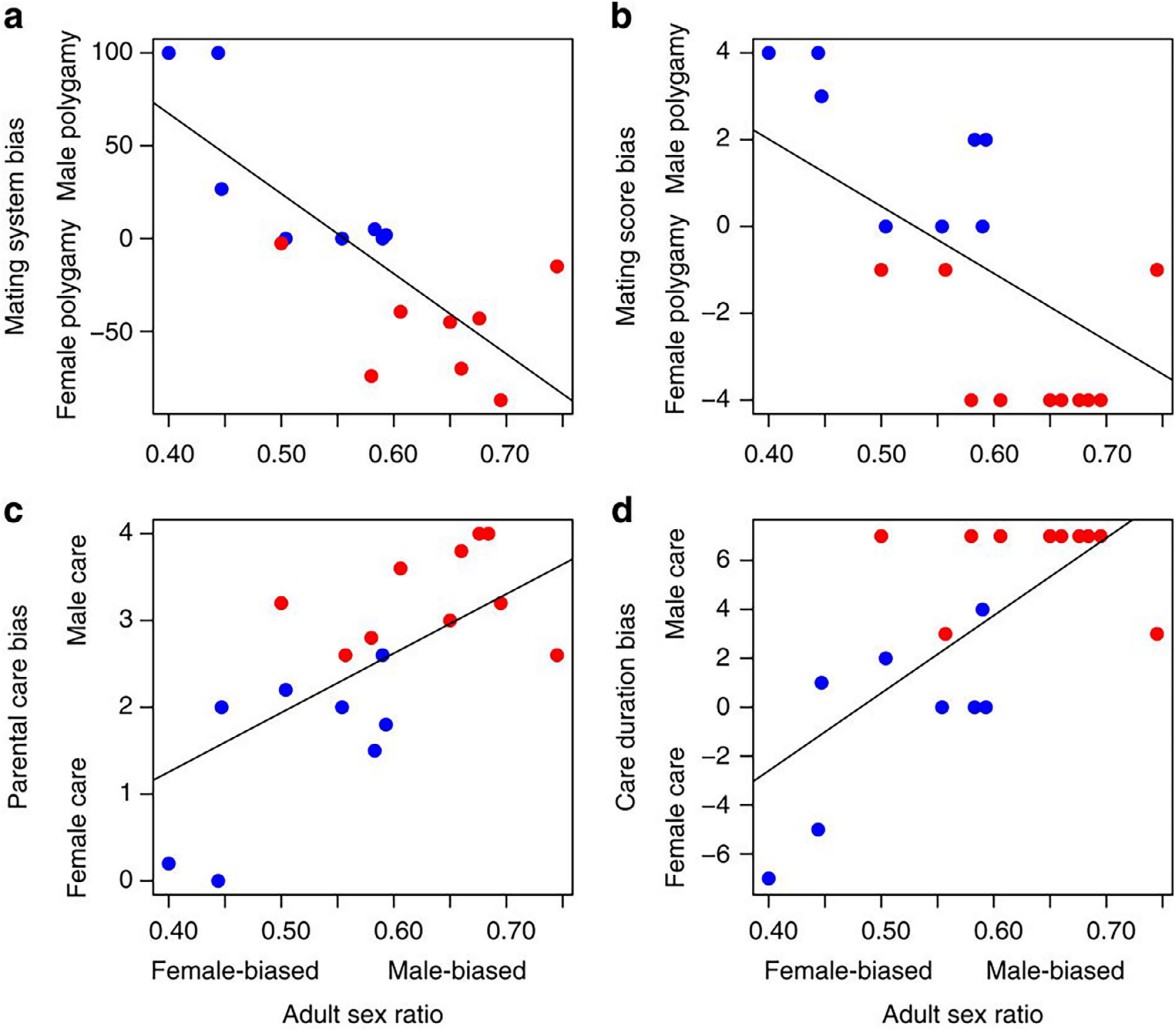
Associations between mating systems, parental care and adult sex ratios. Based on [59] (copyright pending). Blue dots and red dots refer to species with conventional or sex‐role reversed behaviour, respectively.

Comparative analyses then tested whether ASR predicts variation in social systems across species. Among birds, ASR covaries strongly with divorce, social polygamy, and infidelity: the rarer sex is more likely to change mates, whereas the common sex shifts its parental effort and competitive behavior [59, 60, 102]. Follow‐up studies showed that this general pattern holds across a broader range of taxa and, somewhat surprisingly, identified ASR as the strongest predictor of sex differences in avian reproductive strategies [61]. Together, these results established ASR as a robust predictor of sex roles, mating strategies, and parental care (Figure 3). Further conceptual developments emerging from this population‐level approach are summarized in Box 1, “*Offshoots of feedback thinking*.”

Having shown that ASR predicts variation in social systems, the next step was to ask which processes generate ASR itself. Phylogenetic analyses across avian clades demonstrated that sex‐biased adult mortality—rather than hatching or fledging ratios—best explains variation in ASR [60]. Across tetrapods, differences in sex‐determination systems also correspond to systematic ASR skews, revealing how genetic architecture can ripple upward into social organization [62, 63]. Later comparative models linked ASR to sexual selection intensity and sex‐specific maturation rates, showing how the social environment feeds back into life‐history schedules. Across amniotes, ASR correlates with sexual size dimorphism (SSD), with the rarer sex often evolving larger body size to exploit enhanced mating opportunities [64, 65]. Collectively, these results reposited ASR as the demographic hub through which ecology, genetics, and behavior interact [55].

**Box 1. Offshoots of feedback thinking**

The population‐level framework for studying breeding systems inspired two notable extensions of the feedback thinking. Both relate to sexual selection and together illustrate how feedback perspectives can yield new insights into processes that are otherwise considered well understood.

**Sexual selection and Rensch's rule**

Work on shorebirds revealed that sexual selection can help explain Rensch's rule, the macroevolutionary pattern in which male body size scales more steeply than female size across related species [66]. Shorebirds exhibit striking variation in sexual size dimorphism (SSD), and this variation aligns closely with differences in mating systems. In polygamous species, where sexual selection is typically stronger, males and females often differ in size, whereas in monogamous species body sizes are usually similar.

Importantly, the *direction* of dimorphism depends on courtship mode. In aerially displaying species—especially those that perform acrobatic twists and turns during courtship—males are often smaller than females, likely enhancing manoeuvrability during flight displays. In contrast, males in ground‐displaying species tend to be larger, conferring advantages in male–male competition. These contrasting selective regimes—all expressions of sexual selection—appear to generate the allometric relationships consistent with Rensch's rule in shorebirds [34], and more broadly across birds [67, 68].

**The dispersal‐to‐mate hypothesis**

At a macroevolutionary scale, sexual selection is often viewed as an engine of speciation, driving divergence through the rapid evolution of ornamental traits. Comparative work on shorebirds, however, suggested an alternative link between mating systems and speciation: the dispersal‐to‐mate hypothesis.

To breed polygamously, individuals may need to move among distant breeding sites to access multiple mates rather than remaining at a single location. Such mate‐seeking dispersal—common in many shorebirds [18, 69] —promotes gene flow and leads to more panmictic populations. In contrast, monogamous species often breed locally, favoring reduced dispersal, stronger local adaptation, and greater genetic differentiation among populations. Comparative genetic analyses support this hypothesis: polygamous shorebirds exhibit higher gene flow and weaker genetic differentiation across geographically distant populations than their monogamous relatives [58, 70, 71, 72, 100].

**Synthesis**

Together, these spin‐offs demonstrate how feedback thinking at the population level can illuminate macroevolutionary patterns—from allometry to speciation—by tracing how individual behavioral strategies scale up into long‐term evolutionary consequences.

The ASR framework has also enabled tests of long‐standing evolutionary hypotheses. A global comparative analysis found no evidence that gametic investment predicts parental sex roles, whereas both sexual selection and ASR did [73]—redirecting attention from sex differences in gamete size (anisogamy) to population demography as the primary driver of sex differences in social behavior [31, 74].

Despite these advances, a fundamental question has remained: is ASR skew a cause or a consequence of social behavior? On one hand, demographic processes can generate skewed ASRs that subsequently alter mating and care dynamics [56, 60, 63]. On the other hand, social interactions can themselves bias ASR. For example, parental effort may reduce adult survival through the cost of reproduction, while group living may enhance survival via the benefits of sociality [75, 76]. Disentangling these causal directions is challenging because survival patterns, sex ratios and social behavior are tightly intertwined.

Nevertheless, recent phylogenetic path analyses in birds suggest that the former hypothesis—demographic causation—is more consistent with the data than the latter. These analyses indicate that skewed ASRs tend to emerge first within populations and subsequently shape social systems, rather than arising as a downstream consequence of social behavior [105].

Taken together, the studies reviewed above established ASR as the keystone of the demographic feedback model: a single demographic variable that bridges behavior, ecology, and evolution (see also [55]). By replacing the vague concept of “mating opportunity” with a measurable quantity, ASR provides a common language for linking individual decisions to population structure, and population structure to evolutionary outcomes. This integrative role of ASR has now been recognized well beyond the systems in which it was originally developed, with independent syntheses in behavioral ecology, anthropology, and demography converging on ASR as a fundamental determinant of social organization and population viability across taxa, including humans [77, 78, 101]. It anchors the study of sex roles in demographic reality—where genes, ecology, and social behavior continually coevolve through feedback [79].

### 3.3 From Evolutionary Theory to Biodiversity Conservation

Because reproductive behavior is tightly linked to adult sex ratio—and therefore to the core demographic processes that determine population stability—integrating ASR into conservation programmes is not optional but essential. If sex‐biased survival, climate stress, habitat structure, or hunting pressure drive ASR away from balance, the resulting demographic distortion can erode parental cooperation, destabilize pairing systems, and reduce recruitment [80, 81, 82, 83, 104]. Recognizing ASR as both a cause and signal of demographic health thus provides a powerful diagnostic tool for anticipating collapse before it occurs [55]. This logic now underpins several conservation initiatives in which feedback thinking has been applied directly to management [96, 97, 98, 99]. Here, we outline how the feedback framework has been translated from theory into practice across different conservation contexts.

A landmark example is the recovery of the collared pratincole (*Glareola pratincola*) in Hungary. These birds nested in seemingly suitable but intensively farmed fields—an ecological trap in which habitat cues misled breeders into low‐fitness sites [17]. By applying the framework's three‐step logic—(1) quantifying reproductive outcomes, (2) identifying failure points, and (3) intervening at the key trade‐offs—conservation managers modified cultivation practices to improve nesting success. As a result, breeding success increased and population recovery followed [84]. More generally, sex‐structured demography can reveal where ASR is being skewed by mortality, movement, or habitat alteration, enabling managers to stabilize breeding populations before systems shift into low‐fitness reproductive regimes.

Feedback principles may also apply to *ex situ* conservation. A recent global analysis of birth sex ratios in zoos [85] revealed consistent phylogenetic biases: female‐biased births in ungulates, male‐biased in primates, and trait‐linked variation across birds and mammals. These findings show that even in controlled environments, sex‐ratio distortions can emerge along evolutionary lines—often shaped by mating systems, sexual size dimorphism, and brood size. Critically, thirty conservation flagship species exhibited significant birth biases in zoos, including tigers, pygmy hippos, and African wild dogs, raising concerns about the demographic sustainability of some captive populations. Together, these results demonstrate that the demographic feedback shaping wild populations may also operate under human care. Consequently, monitoring ASRs and birth sex ratios should be incorporated into zoo breeding programmes to prevent subtle, long‐term erosion of population viability.

Field programmes designed to test the feedback framework—most notably in Maio (Cabo Verde) and Andavadoaka (south‐west Madagascar)—have evolved into community‐led conservation models [86, 87]. Both initiatives embed evolutionary research on mating systems and parental care within local communities by directly supporting livelihoods. On Maio, long‐term monitoring of plovers contributed to securing Ramsar Convention designation for key wetlands and to the development of coordinated conservation actions and a network of protected sites. In Madagascar, data from plover projects are guiding an 8,000‐hectare Ramsar proposal of Andavadoaka wetlands, supporting the sustainable use and long‐term management of natural resources.

Recursive thinking also extends to education. The Bath‐led team founded local NGOs in both Cabo Verde and Madagascar— the Maio Biodiversity Foundation (FMB) and Madagascar Nature Conservation (MNC), respectively—which integrate field‐based monitoring with environmental education. Evaluations of teaching in local schools showed that education itself can be managed as a feedback process: classroom activities generate hypotheses; research tests them; and conservation outcomes return as teaching material. This cycle builds knowledge and local ownership simultaneously, transforming theory into durable social infrastructure [88].

**FIGURE 4:**
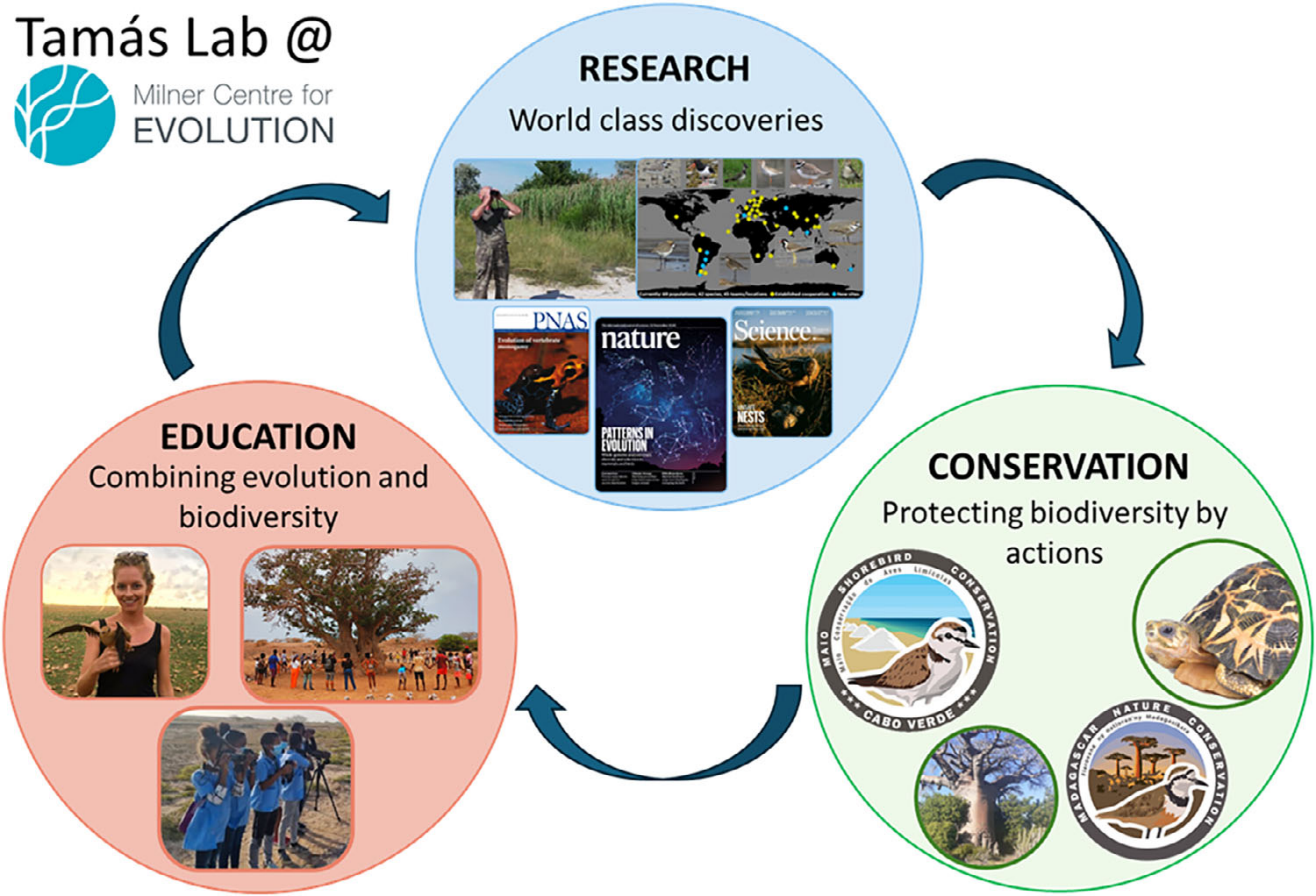
Conceptual structure of the Bath research group.

The Milner Centre for Evolution at the University of Bath has become the nucleus of the demographic–behavioral synthesis (Figure 4). Over two decades, this synthesis matured from a conceptual model into a living framework linking breeding systems, phenotype and population demography. Its logic is inherently recursive: individuals make decisions within an opportunity landscape that their own behavior reshapes. The adult sex ratio provides the integrative measure of that landscape—predicting pair‐bond dynamics, mating systems, sexual selection intensity, maturation timing, and even sexual size dimorphism—while ecological and genetic processes help explain how such shifts arise. Because the framework is clearly defined, measurable, and predictive, it moves seamlessly from theory to population monitoring to wildlife management, representing a rare feedback loop in which scientific insight, conservation practice, and community empowerment reinforce each other.

## 4 Legacy: A Recursive View of Evolution

The Milner Centre for Evolution has provided the steady ground on which these ideas have matured. Although the Centre—and the University of Bath more broadly—hosts only a small number of researchers in organismal biology, it has fostered an environment where intellectual curiosity thrives and excellence is expected. Within this atmosphere, our group developed collaborations that bridged disciplines and continents, working closely with excellent colleagues at Bath, including Matt Wills, Araxi Urrutia, Robert Kelsh, Nick Priest, and Momna Hejmadi. Fully realizing these concepts requires a broad network of partners across the UK and abroad, where complementary expertise, field systems, and theoretical skills converge.

We identify four circumstances were especially influential in shaping this body of work.

First, nearly all projects began from a fundamental curiosity about how nature organizes itself. The social lives of animals pose puzzles that can remain unresolved even after decades of study, and we are proud that some of our efforts have helped illuminate the dynamics of breeding systems, sexual selection, and sexual dimorphism.

Second, a continuous stream of outstanding PhD students—both at Bath and at partner institutions—has been central to this progress. Through their projects, they transformed abstract frameworks into testable questions, producing results that advanced theory while launching their own careers in academia or conservation. The creativity, persistence, and hard work of successive generations of PhD students have been the lifeblood of this enterprise.

Third, the exchange of ideas through international visits often triggered new conceptual turns. For instance, a sabbatical at Harvard University led to the synthesis presented in *Social behavior: genes, ecology and evolution* [89]. A visiting professorship at the University of Groningen catalyzed the development of the sex‐ratio projects [60], while further collaborations at the University of Bielefeld and the University of Göttingen refined the links between sex roles and population demography [56, 60, 61]. These experiences illustrate that intellectual progress—like evolution itself—thrives on movement, recombination and exchange.

Fourth, the science has never remained confined to academia. We have worked deliberately to connect these findings to biodiversity conservation and education. Involving young researchers, senior colleagues and local communities in Madagascar, Cabo Verde, and elsewhere has helped cultivate a conservation mindset rooted in scientific understanding. This engagement continues to nurture new generations of scientists and conservationist practitioners—evidence that the feedback between research and real‐world impact is not merely theoretical, but active and capable of sustaining itself.

## 5 Future Directions and Challenges

The demographic feedback model linking demography, behavior, and evolution is still in an early phase. Its promise lies not only in explaining existing data, but in unifying fields that have long evolved separately. We identify four frontiers where progress is likely to be decisive.

### 5.1 Expanding Field Protocols Across Taxa

Field protocols for estimating ASR and breeding biology have shown how demographic feedbacks can be mapped in wild avian populations [90]. The next step is to extend this approach beyond birds. Comparable, standardized protocols are needed for mammals, reptiles, amphibians, fishes, and invertebrates—including insects and spiders—to test whether feedback dynamics follow general principles or taxon‐specific routes. Only through such cross‐clade field programmes can the recursive structure of evolution be assessed empirically.

### 5.2 Comparative Inference and Causality

Testing feedbacks requires phylogenetic thinking and comparative methods that move beyond one‐way causation. Current phylogenetic path models allow directional testing, but struggle to accommodate reciprocal causality [91]. A new generation of models is therefore needed—ones that can directly contrast unidirectional and feedback hypotheses, and integrate insights from phylogenetic, coevolutionary, and demographic frameworks. Developing this methodological synthesis represents a major frontier for the coming decade in comparative biology.

### 5.3 Modelling Nested Feedbacks

Mathematical models in behavioral ecology increasingly recognize the importance of feedback, yet explicit implementation remains limited [92, 93]. Future models must capture the nesting of feedback loops across scales—how ecological context, gametic investment, demography, and sexual selection interact dynamically. Integrating these layers into game‐theoretical or agent‐based frameworks will be essential to disentangle the web of reciprocal effects shaping sex roles, life histories, and diversification. We are only at the threshold of this new era of nested‐feedback modelling.

### 5.4 From Evolutionary Theory to Conservation and Education

Behavioral ecology has matured to the point where it can meaningfully inform conservation practice. The challenge ahead is to embed feedback thinking within the logic of conservation planning, both in situ and *ex situ*. Demographic tools such as ASR can function as early‐warning indicators of population instability, while evolutionary insights into breeding systems, sexual selection, and life‐history trade‐offs can guide adaptive management. At the same time, these advances must be translated into education and capacity building—training local scientists, managers, and communities to recognize and act upon feedback loops in their own ecosystems. Bridging evolutionary theory and conservation practice is not optional; it is both a scientific and an ethical responsibility.

## Conflicts of Interest

The authors declare no conflict of interest.
